# Serological and cellular response to mRNA-SARS-CoV2 vaccine in patients with hematological lymphoid malignancies: Results of the study “Cervax”

**DOI:** 10.3389/fonc.2023.1133348

**Published:** 2023-02-27

**Authors:** Sara Mohamed, Elisa Lucchini, Francesca Sirianni, Marika Porrazzo, Laura Ballotta, Mario Ballerini, Giovanni Maria De Sabbata, Eleonora De Bellis, Ilaria Cappuccio, Marilena Granzotto, Barbara Toffoletto, Ilaria Fortunati, Anna Russignan, Emilia Elzbieta Florea, Lucio Torelli, Francesco Zaja

**Affiliations:** ^1^ UCO Ematologia, Azienda Sanitaria Universitaria Giuliano Isontina, Trieste, Italy; ^2^ SC Laboratorio Analisi, Azienda Sanitaria Universitaria Giuliano Isontina, Trieste, Italy; ^3^ Dipartimento di Scienze Mediche, Chirurgiche e della Salute, University of Trieste, Trieste, Italy; ^4^ Dipartimento di Medicina, sezione Ematologia, University of Verona, Verona, Italy

**Keywords:** hematological malignancies (HM), COVID-19, m-rna vaccine, chronic lymphocitic leukaemia, lymphomas, multiple myeloma

## Abstract

messenger RNA (mRNA)-Severe acute respiratory syndrome coronavirus 2 (SARS-CoV2) vaccines such as BNT162b2 became available in late 2020, but hematological malignancy patients (HM pts) were not evaluated in initial registration trials. We hereby report the results of a prospective, unicentric, observational study Response to COVID-19 Vaccination in hEmatological malignancies (CERVAX) developed to assess the postvaccine serological and T-cell-mediated response in a cohort of SARS-CoV2-negative HM pts vaccinated with BNT162b2. Patients with lymphomas [non-Hodgkin lymphoma (NHL) and Hodgkin lymphoma (HL)], chronic lymphocytic leukemia (CLL), and multiple myeloma (MM); off-therapy for at least 3 months; in a watch-and-wait program; or in treatment with ibrutinib, venetoclax, and lenalidomide were included. Different time points were considered to assess the serological response to the vaccine: before the second dose (T1), at 3–6–12 months after the first dose (T2–3–4, respectively). Since March 2021, 39 pts have been enrolled: 15 (38%) NHL, 12 (31%) CLL, and 12 (31%) MM. There were 13 of the 39 pts (33%) seroconverted at T1; an increase of the serological response was registered after the second dose (T2) (22/39 pts, 56%) and maintained after 6 months (22/39 pts, 56%) and 12 months (24/39 pts, 61%) from the first dose (T3–T4, respectively). Non-serological responders at T4 were 7/39 (18%): 0/15 NHL, 1/12 MM (8%), and 6/12 CLL (50%). All of them were on therapy (one lenalidomide, three ibrutinib, and three venetoclax). SARS-CoV2-reactive T-cell analysis (interferon gamma release assays) was available since June 2022 and was evaluated at 12 months (T4) from the first dose of vaccine in 31/39 pts (79%). T-cell-mediated-responders were 17/31 (55%): most of them were NHL and MM (47%, 41% and 12% for NHL, MM, and CLL, respectively). Both serological and T-cell non-responders were represented by pts on active therapy (venetoclax/ibrutinib). During the period of observation, eight (20.5%) pts developed mild SARS-CoV2 infection; no coronavirus disease 19 (COVID-19)-related deaths or hospitalizations were registered. In conclusion, in our cohort of lymphoproliferative pts receiving BNT162b2, CLL diagnosis and venetoclax/ibrutinib seem to be related with a lower humoral or T-mediated response. Nevertheless, the efficacy of mRNA vaccine in HM pts and the importance to continue the vaccine program even in non-responders after the first dose are supported in our study by demonstrating that a humoral and T-cell-mediated seroconversion should be observed even in the subsets of heavily immunocompromised pts.

## 1 Introduction

The effects of the new coronavirus SARS-CoV2-19 infection (COVID-19) significantly affected the prognosis of hematological malignancy patients (HM pts) with a COVID-19-related mortality rate that, particularly in the initial phase of the pandemic, reached up to 35%–40% (1, 2). The status of immunodeficiency secondary to the disease itself or treatment lead HM pts to be particularly prone and vulnerable to develop severe COVID-19 infections and their related complication. For these reasons, the attempt to protect these patients from SARS-CoV2-19 is critical.

Based on the results of the randomized phase III clinical trials, the mRNA vaccines BNT162b2 (Pfizer/Biontech) and mRNA-1273 (Moderna) became available from the late 2020 revealing a proper safety and efficacy profile in healthy subjects (3). However, HM pts were not included in these trials, and vaccine efficacy in this group could not be therefore evaluated. The profound and lasting suppression of B and T immune compartments with severe hypogammaglobulinemia and CD4-T lymphocytopenia make HM pts generally poor responders to different type of vaccines (4, 5). As far as SARS-CoV2-19 vaccinations are concerned, preliminary studies exploring the assessment of the response to BNT162b2 in HM pts demonstrated a low seroconversion rate after the first dose, ranging from 18% to 25% (6, 7). Furthermore, the activity of neutralizing antibodies and the level and timing of the T-cell response are still poorly known in these subsets of patients. On these grounds, it is important to identify the variables that may influence the immune response to the vaccines in order to assess the most appropriate strategy for different subsets of HM pts.

We here report the results of a prospective study (CERVAX) aimed to evaluate the humoral and T-cell mediated response to BNT162b2 vaccination in a selected cohort of HM pts affected by lymphoproliferative diseases who were followed in a single institution and the impact that vaccination had in the development of COVID-19 infection.

## 2 Patients, materials, and methods

### 2.1 Design of the study and ethical considerations

CERVAX is a prospective observational study conducted at the Department of Hematology of the Trieste University Hospital. The study started in March 2021, and the last evaluation was conducted in July 2022 after having received the approval from the Ethic Committees of the Spallanzani Hospital in Rome and the Regione Friuli Venezia Giulia. This study was conducted in agreement with Helsinki Declaration of 1975, as revised in 2008, and all patients received and signed an informed consent prior to participation in the study.

### 2.2 Patients

Patients aged ≥18 years old, diagnosed with non-Hodgkin lymphoma (NHL) or Hodgkin lymphoma (HL), chronic lymphocytic leukemia (CLL), and multiple myeloma (MM) were eligible for the study. Subjects who had previously developed documented COVID-19 infection were excluded. All patients underwent to BNT162b2 mRNA-COVID-19 vaccination. Among the inclusion criteria, it was mandatory to be off anti-HM therapy for at least 3 months or to be in a watch-and-wait condition or, in treatment with bruton tyrosine kinase (BTK)-inhibitors (ibrutinib) or B-cell lymphoma 2 (BCL-2) inhibitors (venetoclax) without rituximab (R), or immunomodulatory drugs (IMIDs) (lenalidomide) (with or without dexamethasone but in the absence of immunotherapeutic agents, e.g., daratumumab). The use of chronic oral therapies given for palliative purposes, such as chlorambucil, was not an exclusion criterion.

### 2.3 Aims of the study

The primary endpoint of the study was to evaluate the immunological (serological and T-cell-mediated) response in HM pts’ anti-SARS-CoV2 vaccination using BNT162b2 mRNA vaccines.

The secondary endpoints were to assess the time of acquisition and maintenance of immunity 12 months after initial vaccination; to identify immunological response patterns in the different diseases considered in the study and in relation to the immunological characteristics of patients assessed at the baseline; to assess the impact of the therapies used in the HM pts on the development of postvaccination immunity; to monitor postvaccination side effects; and to assess the incidence of SARS-CoV2 infection since vaccine administration.

### 2.4 Vaccination

The vaccine schedule used consisted of two doses of the BNT162b2 mRNA-COVID-19 vaccine 21–28 days apart. During the study, after the agenzia italiana del farmaco (AIFA) approval of the third dose of vaccine, all patients received the booster dose from October 2021 as recommended by the health authorities.

### 2.5 Immune response evaluation: Timing and methodology for the evaluation of serological and T-cell-mediated response

The medical history, blood cell count, and differential dosages of IgG, T-CD4+, T-CD8+ cells, and IgG anti-SARS-CoV2 were evaluated at the baseline (T0). The following time points were planned to assess the serological response to the vaccine: before the second dose (T1) and 3, 6, and 12 months after the first dose (T2, T3, and T4, respectively). The qualitative and quantitative determination of anti-SARS-CoV-2 IgG antibodies was performed by an automated two-step immunoassay in human serum and plasma samples using chemiluminescent microparticle capture immunoassay technology (8). The SARS-CoV-2 IgG II Quant assay (8) was the tool utilized to evaluate the serological response. This test is designed to detect immunoglobulin class G (IgG) antibodies directed against the receptor-binding domain of the S1 subunit of the SARS-CoV-2 spike protein in serum and plasma (9–11).

In order to evaluate the T-cell response to SARS-CoV2, an interferon gamma (IFN-γ) release assay (IGRA) test was adopted 12 months after the first dose of the vaccine. This is an enzyme-linked immunosorbent assay for the quantitative detection of IFN-γ production *in vitro* by T cells in response to the stimulation with the SARS-CoV-2 spike (12).

### 2.6 Statistical analysis

A descriptive analysis of the demographic and clinical characteristics of the patients was performed by using median and range for continuous variables and absolute and relative frequencies for categorical variables. Correlations between groups of patients were made by the χ2 test or by the exact Fisher test, as appropriate, at the significance level of alpha = 0.05. All calculations were made using software R, package Rcmdr (version 2.8-0).

## 3 Results

### 3.1 Patients’ characteristics

From March 2021 to July 2021, 39 pts were enrolled: 15 (38%) NHL, 12 (31%) CLL, and 12 (31%) MM. Clinical, histotype, and biologic features are summarized in **Table 1**. Briefly, men and women were equally distributed (51% vs. 49%, respectively). The median age of the entire cohort was 77 years (range 52–88 years). There were 23 (59%) patients who were not receiving any therapy at the time of the enrolment, whereas 16 (41%) were currently on medication: ibrutinib in 4/16 (25%), venetoclax in 5/16 (31%), and lenalidomide in 7/16 (44%), respectively. Most of the patients previously received more than one line of treatment (77%). One NHL pt did not receive any previous treatment, while the remaining 14 pts (93%) had a median of one previous line (range 1–4), consisting of a rituximab (R)-based protocol (R alone, R-CHOP, and R-CVP). There were 9 out of 12 CLL pts (75%) who received a median of two previous lines (range 1–4) (FCR, R-chlorambucil, R alone, R-bendamustine, idelalisib, ibrutinib, and venetoclax). There were 7 out of 12 MM pts (58%) who had a median of one previous line (range 1–4) (bortezomib-based protocols: VTD, VD, VMP, and VCD; carfilzomib and lenalidomide). There were 36 out of 39 pts (93%) who presented a stable response of the disease.

**Table 1:** Clinical, histological, and biological patients’ features.

Features (tot. Pts 39)	N (%)
Gender	
F	19 (49)
M	20 (51)
**Age (median) [range]**	77 [52-88]
F	81 [52-87]
M	76 [52-88]
Hematological Malignancies	
NHL	15 (38)
- Marginal-zone lymphoma	3 (20)
- Follicular lymphoma	5 (33)
- Hairy-cell leukemia	1 (7)
- Lymphocytic lymphoma	1 (7)
- DLBCL	4 (26)
- Mantle-cell lymphoma	1 (7)
CLL	12 (31)
MM	12 (31)
Therapy	
On therapy (ibrutinib, venetoclax, and IMIDs)	16 (41)
None	23 (59)
Previous therapy lines	
0	9 (23)
≥1	30 (77)
Disease status (T0)	
CR, PR, SD, and VGPR	36 (92)
PD	3 (8)
IgG status at T0	
<500 mg/dl	11 (28)
≥500 mg/dl	28 (72)
CD4+ at T0	
<400/mmc	16 (41)
≥400/mmc	23 (59)
CD8+ at T0	
<400/mmc	16 (41)
≥400/mmc	23 (59)
Status at last follow-up	
Alive	38 (97)
Dead	1 (3)
Adverse events (CTCAE v 5.0)	
Grade 0	31 (79.5)
Grade 1	8 (20.5)
Grade ≥2	0

The baseline levels of IgG < 500 mg/dl and the number of T-CD4+ and/or T-CD8+ < 400/mmc were observed in 11 (28%), 16 (41%), and 16 (41%) patients, respectively. The median period of observation from T0 was 14.2 months (range 12.1–16.5 months).

### 3.2 Humoral and T-cell response to SARS-CoV2 BNT162b2 mRNA-COVID-19 vaccine

**Figure 1:**
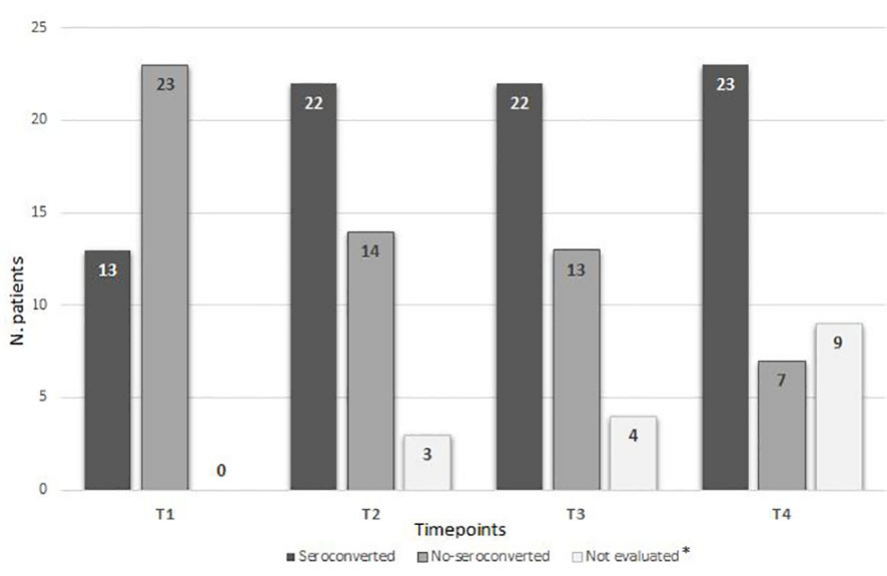
Seroconversion according to time points. * Due to an ongoing COVID-19 infection during the study or lost to follow-up.

As shown in **Figure 1** and **Table 2**, a serological response was observed in 13 (33%) patients at T1 and in 22 patients (56.4%) at T2. The rate of response was maintained after 6 (T3: 22 patients) and 12 months (T4: 24 patients) from the first dose in 56.4% and 61%, respectively. During the 12-month observation period, the third and the fourth doses of the mRNA-SARS-CoV2 vaccine were available and recommended for all patients participating in the study. Three seroconversions were observed after the third dose (1 pt MM; 2 pts CLL), while two were registered after the fourth (1 pt MM; 1 pt CLL). When analyzed according to the subtype of HMs, the highest response rate was observed in NHL and MM subgroups, 73.3% and 75% respectively, while the lowest was observed in CLL (33.3%). Non-responders at T4 were 7 (18%) pts: 0/15 NHL, 1/12 MM (8%), and 6/12 CLL (50%). All the non-responders were on therapy (one lenalidomide, three ibrutinib, and three venetoclax). Median antibody anti-SARS-COV2 levels according to the time point and HM are summarized in **Table 3**. SARS-CoV2-reactive T-cell analysis (IGRAs) was available in our hospital since June 2022 and was evaluated at 12 months (T4) from the first dose of vaccine in 31 pts (79%). A T-cell response was documented in 17/31 pts (55%) (**Table 4A**); 8/17 (47%) NHL pts, 7/17 (41%) MM pts, and 2/17 (12%) CLL pts. Only 5/17 (30%) T-cell responders were on therapy (one venetoclax and four lenalidomide), whereas most were off therapy (12/17 pts: 70%). Non-T-cell responders are shown in **Table 4B**: most of them were CLL pts (8/14 pts: 58%). There were 8 of the 14 non-T-cell responders (58%) who were on active therapy: two lenalidomide, three venetoclax, and three ibrutinib.

**Table 2:** Seroconversion according to hematological malignancy (HM).

Hematological Malignancies	N(%)	T1 seroconverted (%)	T2 seroconverted (%)	T3 seroconverted (%)	T4 seroconverted (%)
NHL	15 (38)	7 (46,6)	12 (80)	12 (80)	11 (73,3)
CLL	12 (31)	0	1 (8)	2 (16,6)	4 (33,3)
MM	12 (31)	6 (50)	9 (75)	8 (66,6)	9 (75)
Total	39	13 (33)	22 (56,4)	22 (56,4)	24 (61)

**Table 3:** Median level of antibody anti-SARS-CoV2 according to time points and HM.

Median level of antibody anti-SARS-CoV2 [positive if >50 AU/ml]				
Hematological Malignancies	T1 (N [range])	T2 (N [range])	T3 (N [range])	T4 (N [range])
NHL	39.7 [0.5–749.1]	890 [1.7–3.866]	472.5 [0–19,582]	4,377 [289–144,148]
CLL	0.95 [0–7.2]	0 [0–502]	0 [0–422]	769 [0–88,000]
MM	64.6 [0–4,271.2]	913 [0–11,136]	424 [0–8,700]	5,494 [0–73,665]

**Table 4A:** T-cell-mediated responders (IGRAs) combined to HM and according to therapy.

Hematological Malignancies/IGRAs positive	N(%)	Off therapy (N [%])	On therapy (N [%])
NHL/IGRAs +	8 (47)	7 (87)	1 (13)
CLL/IGRAs +	2 (12)	1 (50)	1 (50)
MM/IGRAs +	7 (41)	4 (57)	3 (43)
Total	17	12 (70)	5 (30)

**Table 4B:** Non-T-cell mediated responders (IGRAs) combined to HM and according to therapy.

Hematological Malignancies/IGRAs negative or indeterminated	N(%)	Off therapy (N [%])	On therapy (N [%])
NHL/IGRAs – or indeterminated	3 (21)	3 (100)	0
CLL/IGRAs – or indeterminated	8 (58)	2 (25)	6 (75)
MM/IGRAs- or indeterminated	3 (21)	1 (33)	2 (66)
Total	14	6 (42)	8 (58)

The results of seroconversion combined to IGRAs are shown in **Table 5**. Considering the exploratory nature of our study and the small number of cases, no correlation between IgG, T-CD4+/CD8+ values at T0, and seroconversion was found (p-value 0.25 and 0.48, respectively). Seropositivity was associated to a T-cell mediated response in 16 (52%) of the patients analyzed: NHL and MM registered a higher rate (73% and 70%, respectively). The lack of a T-cell response, despite the occurrence of an antibody response, was observed in seven (23%) patients with no distinction according to the subgroup of HM (**Table 5**). As expected, the subgroup with both seronegativity and no T-cell response was mainly represented from CLL pts (4/10, 40%).

**Table 5:** Seroconversion/T-cell mediated response (IGRAs) in HM.

	tot(%)	NHL (11 pts) [%]	MM (10 pts) [%]	CLL (10 pts)[%]
Sero-resp +/IGRA +	16/31 (52)	8/11 (73)	7/10 (70)	1/10 (10)
Sero-resp -/IGRA +	1/31 (3)	0/11	0/10	1/10 (10)
Sero-resp +/IGRA -	7/31 (23)	2/11 (18)	2/10 (20)	3/10 (30)
Sero-resp -/IGRA -	5/31 (16)	0/11	1/10 (10)	4/10 (40)
Sero-resp + or -/IGRA indeterminated	2/31 (6)	1/11 (9)	0/10	1/10 (10)

### 3.3 Safety data

During the period of observation from the beginning of the study (the median time of observation 14.2 months), 8/39 (20.5%) patients developed an adverse event graded 1 according to Common Terminology Criteria for Adverse Events (CTCAE) v5.0 with most symptoms being represented by fever, myalgia, and local pain at the vaccine inoculation site (**Table 1**). Symptoms were registered after both the first and second dose of vaccination.

### 3.4 Development of COVID-19 infections

During the study period, eight (20.5%) patients had COVID-19 infection; each of them developed COVID infection after 2.3, 6.2, 6.3, 12.2, 12.3, 13.1, 13.4, and 13.5 months, respectively, from the second dose (four non-seroconverted and four seroconverted). Three pts developed COVID-19 infection during September–November 2021, while five pts had a breakthrough infection between May–July 2022. The COVID-19 variant involved in the reported infections was not determined. According to the Italian epidemiological data, the predominant COVID-19 variants in these periods were Delta and Omicron. In all cases, the disease course was mild: no COVID-19-related deaths were recorded, and none of patients required hospitalization. Only one patient died due to a progression of his disease (mantle cell lymphoma) after recovering from COVID infection.

## 4 Discussion

Our study confirms that, in patients with lymphoproliferative disorders, the response to SARS-CoV2 vaccine is lower than expected in the healthy population; the mRNA-SARS-CoV2 vaccine has been proven safe, and no severe COVID-19 infections were registered.

The effects of mRNA-SARS-CoV2 vaccines in HM pts have already been evaluated in a multicenter European study suggesting a protective role with a reduction in the mortality rates (from 31% to 12.4% comparing the pre- and postvaccination availability) (13). As noted, several factors play a role to determine a lower immune response in HM pts to general vaccines and SARS-CoV2 vaccines. The immunogenicity to mRNA-SARS-CoV2 vaccines in HM pts has been explored in several studies (14–17) focusing mainly on the humoral response, while, up to now, only few data are available as far as the T-cell mediated response is concerned. Malard et al. (17) reported a T-cell response in 53% (36/58 pts) of patients with myeloid or lymphoid malignancies; notably, the T-cell response to the mRNA-SARS-CoV2 vaccine was also observed in those pts who had no humoral response. However, Mairhofer et al. (18) and Ehmsen et al. (19) reported frequent impaired humoral and cellular responses in HM pts after two doses of the mRNA-SARS-CoV2 vaccine.

Many variables may influence the response to SARS-CoV2 vaccines in HM pts, among which are the subgroup of HMs, the type of treatments, and the timing of vaccination. In patients with lymphoproliferative disorders, the humoral response after two doses of mRNA vaccine was registered from 40% in CLL to 50% in lymphomas, respectively (20–24). The response to vaccination after anti-CD20 was generally negligible and related with the timing of treatment (from 0% to 10% to 25% to 35% in those patients who received anti CD20 before or after 12 months, respectively) (21). BTK-I (ibrutinib) and BCL2-I (venetoclax) resulted to be associated with a low humoral response after two doses of mRNA vaccine (0%–18% and 0%–24%, respectively) (22, 23). Few data are still available correlating the T-cell response to treatments in lymphoproliferative diseases (25). As far as MM pts are concerned, a lower response to the SARS-CoV2 vaccine has been resulted to be associated with the advanced phase of disease while, on the contrary, no significant relationship was found between seropositivity and the types of treatments, probably due to the different heterogenous therapies available. Van Oekelen et al. (26) reported a seropositivity rate of 68% (219/230 pts) in MM pts after two doses of the mRNA vaccine, but almost 19% of the population analyzed had COVID-19 before the vaccination.

In our study, we focused on a particular selected HM population (NHL, CLL, and MM) in order to explore both humoral and T-cell mediated immune responses to the mRNA-COVID-19 vaccine. We also tried to evaluate the influence of BTK-I and BCL2-I in the immune vaccination response. Despite the limit of a small cohort, our data show both better humoral and T-cell mediated responses in NHL and MM subgroups and confirm the lower B- and T-cell responses in CLL pts, as already reported in literature (22–24). We underline that, in NHL pts, the median time from the last therapy (including anti-CD20, chemotherapy, and/or radiotherapy) was 36 months (range 3–216 months). Anti-CD20 was previously administered in 12/15 NHL pts; 3 of them received anti-CD20 during the 12 months before the vaccination program started, 2 showing seroconvertion.

The humoral response was maintained after the second dose through the 12 months of observation in almost 60% of the entire cohort. This result may have also been influenced by performing the booster dose during the study, which may have prolonged the maintenance of the humoral response. Moreover, our report confirms the role of venetoclax/ibrutinib in reducing the humoral response to the SARS-CoV2 vaccine, as already mentioned in other studies, and suggests an influencing role in affecting the T-cell response.

In addition, in our cohort, a good tolerance of mRNA-SARS-CoV2 vaccines was observed, with only 8/39 patients experiencing grade 1 according to CTCAE v5.0 postadministration side effects, providing an evidence of the good safety profile. It was worth noting that no fatal cases related to COVID-19 infection or fatal adverse events have been observed during the study, supporting the effectiveness of the mRNA-SARS-CoV2 vaccine in protecting HM pts.

In conclusion, our study supports the efficacy and safety of the mRNA-SARS-CoV2 vaccine in HM pts, demonstrating the seroconversion and a T-cell-mediated response even in the subsets of heavily immunocompromised pts and the importance to continue the vaccine program even in non-responders after the first dose. These findings, thus, warrant further confirmations in extended cohorts.

## Data availability statement

The raw data supporting the conclusions of this article will be made available by the authors, without undue reservation.

## Ethics statement

The studies involving human participants were reviewed and approved by Ethic Committees of the Spallanzani Hospital in Rome and the Regione Friuli Venezia Giulia. The patients/participants provided their written informed consent to participate in this study. Written informed consent was obtained from the individual(s) for the publication of any potentially identifiable images or data included in this article.

## Author contributions

FZ and SM designed the study. FZ and SM wrote the article. BT and IF performed all laboratory analyses. LT statistically analyzed the data. All other authors contributed to data collection and data analysis, critically revised the article, and approved the final version of the paper. All authors contributed to the article and approved the submitted version.
